# Abnormal multisensory integration in relapsing–remitting multiple sclerosis

**DOI:** 10.1007/s00221-022-06310-0

**Published:** 2022-01-30

**Authors:** Serena Giurgola, Carlotta Casati, Chiara Stampatori, Laura Perucca, Flavia Mattioli, Giuseppe Vallar, Nadia Bolognini

**Affiliations:** 1 Department of Psychology and NeuroMI, University of Milano-Bicocca, Piazza dell’Ateneo Nuovo 1, 20126 Milan, Italy; 2 Neuropsychology Laboratory, IRCCS Istituto Auxologico Italiano, Milan, Italy; 3 Neurorehabilitation Sciences, IRCCS Istituto Auxologico Italiano, Milan, Italy; 4 Neuropsychology Unit, Spedali Civili of Brescia, Brescia, Italy

**Keywords:** Multisensory integration, Multiple sclerosis, Temporal binding window, Sensory processing, Auditory, Visual

## Abstract

Temporal Binding Window (TBW) represents a reliable index of efficient multisensory integration process, which allows individuals to infer which sensory inputs from different modalities pertain to the same event. TBW alterations have been reported in some neurological and neuropsychiatric disorders and seem to negatively affects cognition and behavior. So far, it is still unknown whether deficits of multisensory integration, as indexed by an abnormal TBW, are present even in Multiple Sclerosis. We addressed this issue by testing 25 participants affected by relapsing–remitting Multiple Sclerosis (RRMS) and 30 age-matched healthy controls. Participants completed a simultaneity judgment task (SJ2) to assess the audio-visual TBW; two unimodal SJ2 versions were used as control tasks. Individuals with RRMS showed an enlarged audio-visual TBW (width range = from − 166 ms to + 198 ms), as compared to healthy controls (width range = − 177/ + 66 ms), thus showing an increased tendency to integrate temporally asynchronous visual and auditory stimuli. Instead, simultaneity perception of unimodal (visual or auditory) events overall did not differ from that of controls. These results provide first evidence of a selective deficit of multisensory integration in individuals affected by RRMS, besides the well-known motor and cognitive impairments. The reduced multisensory temporal acuity is likely caused by a disruption of the neural interplay between different sensory systems caused by multiple sclerosis.

## Introduction

Multisensory integration reflects the ability of synthetizing information from different senses (Bolognini et al. 2013), a function that is based on dedicated cortical and subcortical neural mechanisms (Driver and Noesselt 2008; Stein and Meredith 1993). Multisensory integration is inherently adaptive, optimizing perception, action and cognition (Driver and Noesselt 2008; Stein and Meredith 1993). Indeed, impairments of multisensory integration may disrupt optimal brain functioning (Bolognini et al. 2013; Van der Stoep et al. 2019).

Efficient multisensory integration can be reliably assessed using behavioral paradigms measuring the optimal temporal interval within which the binding of multisensory inputs occurs, the so-called Temporal Binding Window (TBW; e.g., Diederich and Colonius 2009; Stevenson et al. 2016), whose width offers a reliable behavioral marker of multisensory integration efficiency. As a matter of fact, a main feature of multisensory integration is its reliance on temporal factors, whereby sensory stimuli occurring in closed temporal proximity interact at the neural level, enhancing neuronal responses (Bolognini et al. 2005a; Stein and Meredith 1993). Hence, there is a ‘window’ of time within which multisensory stimuli are highly likely to be bound. The multisensory TBW represents an important component of our perceptual experiences, allowing statistical inferences about the likelihood that multisensory stimuli originate from the same event (Wallace and Stevenson 2014).

A standard behavioral paradigm to measure the TBW is the 2-alternative forced-choice Simultaneity Judgement (SJ2; Van Eijk et al. 2008). During the SJ2 task, participants are required to judge whether two stimuli of different sensory modalities, presented with different temporal delays (Stimulus Onset Asynchronies, SOAs), are perceived as simultaneous or not. When observers judge the perceived simultaneity of events separated by a variable SOA, a peaked function of SOA is revealed. The width of that function estimates the TBW, that is, the tolerance of temporal mismatch (i.e., temporal acuity of the simultaneity judgment; Stecker 2018). Additionally, the SJ2 task allows the determination of individual points of subjective simultaneity (PSS), namely the exact point in time at which an individual is most likely to perceive two inputs of different sensory modality as synchronous. The TBW and PSS assess precision and accuracy of multisensory temporal perception (Love et al. 2013).

Differences in TBW width may affect the construction of perceptual, and even cognitive, representations (Tagini et al. 2020; Wallace and Stevenson 2014): multisensory processes form the building blocks upon which perceptual and cognitive representations are created, which depend on the integrity of the information within the incoming sensory streams. Emerging evidence shows that changes in multisensory temporal processes may have cascading effects upon the information processing hierarchy, ultimately impacting on cognitive domains such as attention, executive functions, language, communication, and social interactions (Wallace and Stevenson 2014).

Indeed, a defective (i.e., enlarged) multisensory TBW seems to play a role in the perceptual and cognitive weaknesses featuring some neurological (e.g., Bolognini et al. 2013, 2016; Maccora et al. 2020) and neuropsychiatric/neurodevelopmental diseases, such as autism, dyslexia, schizophrenia (Wallace and Stevenson 2014), as well as in mild cognitive impairment (Chan et al. 2015). On a broader perspective, multisensory integration deficits have been revealed in diseases affecting brain functioning and they have been proposed to be associated, and in some cases could be even responsible of, the clinical symptomology (e.g., obesity, acquired-brain injury, migraine and Parkinson’s and Alzheimer diseases; Bolognini et al. 2013; Brighina et al. 2015; Scarpina et al. 2016, 2019; Tagini et al. 2020; Van der Stoep et al. 2019; Yakubovich et al. 2020; Wu et al. 2012). This because many crucial cognitive and perceptual functions depend on the ability to process and integrate multisensory information. Indeed, multisensory processing provides a more reliable representation of the surrounding environment and optimizes perception and attention, in turn facilitating behavioural responses (Driver et al. 2008; Stein et al. 1993), but also influencing affective and cognitive processes (Wallace et al. 2020). The strong relationship between perception/cognition and multisensory integration has been well-documented by studying the benefits brought about by multisensory stimulations and trainings. For example, spatially and temporally coincident multisensory stimulations can improve spatial orienting and sensory detection in everyday life (Bolognini et al. 2005a, b, 2013; Diederich et al. 2007; Lippert et al. 2007), and multisensory integration influences cognitive development since childhood (Dionne-Dostie et al. 2015), as well as cognitive functioning throughout the lifespan, facilitating time processing and numerical abilities (Bahrick et al. 2000; Jordan et al. 2008), attention, memory and language (Bahrick et al. 2000; 2002; 2018; Calvert et al. 2000; Wallace et al. 2020; for a recent review, see Wallace et al. 2020). Some models even posit that multisensory processing drives the acquisition of higher order cognitive functions (e.g., Bahrick et al. 2002; Bremner et al. 2012). Accordingly, it has been proposed that multisensory integration disorders may negatively impact on mental functioning (Cascio et al. 2016; Van der Stoep et al. 2019).

So far, it is unknown whether demyelinating disorders, in particular Multiple Sclerosis (MS), may disrupt multisensory integration, with an impact on clinical symptoms; some recent evidence is suggestive in this regard (Nava et al. 2018). MS is an immune-mediated disease featured by an abnormal immune response targeting the central nervous system, in turn determining both axonal demyelination and neuronal loss (Manca et al. 2018). Although in the past the investigation of cognitive deficits in MS have been sometimes neglected, nowadays, there is a large consensus about the presence of cognitive decline in this disease, with functional repercussions in daily living. The cognitive profile of MS is characterized by high variability during the disease progression, which is associated to widespread alterations in brain neural networks, with the grey matter lesions representing a main cause of cognitive deficits (Benedict et al. 2020). The cognitive deficits comprise impairments of speed processing, learning, memory, executive functions, and visuospatial processing (Benedict et al. 2020). Even though MS is known to affect sensorimotor processes (Chiaravallotti and De Luca 2008; Nava et al. 2018), a potential concurrent disruption of multisensory integration abilities still needs empirical evidence.

Considering the pathophysiology of MS, and the concurrent cognitive disorders, here we explore whether MS may also disrupt multisensory integration, also considering possible associations with the neuropsychological deficits. We hypothesized that a disconnection syndrome like MS (Manca et al. 2018), featured by processing speed decline as a main cognitive deficit (Van Schependom et al. 2015), could disrupt the functional interplay between different sensory systems, in turn impairing the ability to efficiently process and integrate multisensory stimuli, especially in the temporal domain. If this is the case, MS should be characterized by an enlarged TBW that would reflect a low temporal acuity in multisensory perception.

In the present study, only individuals affected by relapsing–remitting MS (RRMS) were included to recruit a homogeneous group of participants with MS. Indeed, progressive MS is known to be characterized by more pronounced degenerative—rather than inflammatory—pathological changes in the central nervous system, as compared to RRMS (Geurts et al. 2008); moreover, individuals with progressive MS are reported to be more severely impaired in cognition than those with a relapsing–remitting disease (Ruano et al. 2017). The multisensory, audio-visual, TBW of participants with RRMS was compared to that of neurological healthy individuals.

## Materials and methods

### Participants

Twenty-five participants with RRMS (mean age = 49.12 years ± standard deviation = 9.46; females = 15; years of education = 12.44 ± 3.74; time elapsed from MS diagnosis = 14.08 ± 8.6 years; see Table 1) and 30 neurological healthy controls with no history of neurological/psychiatric disorders (mean age = 49.43 ± 7.79 years; females = 19; years of education = 12.73 years ± 2.49) entered this study. Twenty-two participants with RRMS were right-handed, 3 left-handed; similarly, 3 healthy controls were left-handed.

Individuals affected by RRMS were enrolled in the Department of Neurorehabilitation Sciences of IRCCS Istituto Auxologico Italiano (Milan, Italy) and in the Neuropsychology Unit of Spedali Civili of Brescia (Brescia, Italy). The control group of healthy participants was recruited outside the clinical institutes. This multicenter study was approved by the Ethical Committee of the IRCSS Istituto Auxologico Italiano (Protocol n° 25C721), which was the promoting and principal investigator center, and it was conformed to the Declaration of Helsinki (BMJ 1991). All participants were naïve both to the experimental procedure and to the purpose of the study and provided written informed consent to the protocol prior to testing.

Inclusion criteria for participants with RRMS were: (1) being affected by RRMS with an Expanded Disability Status Scale (EDSS) score equal or lower than 8 (Kurtzke 1983); (2) absence of moderate/severe paresis in the upper limbs (Medical Research Council scale > 4/5); (3) absence of visual field defects in both monocular and binocular fields, as assessed with the confrontation visual field testing or, if available, with a computerized visual field perimetry; (4) absence of clinically measurable hypoacusis, assessed using the diapason and Rinne Weber test or, when available, with an audiometric exam; (5) absence of a neurological relapse in the previous 6 months; (6) no history of previous psychiatric/neurological illness before RRMS diagnosis; (7) absence of cognitive decline (i.e., score > 24 at the Mini Mental State Examination, MMSE; Italian normative data from Grigoletto et al. 1999).

For the healthy control group, the inclusion criteria were: (1) no history of previous psychiatric/neurological illness; (2) absence of sensory/motor impairment.

All participants with RRMS underwent a neuropsychological assessment using the Italian version of the Rao’s Brief Repeatable Battery of Neuropsychological Tests (BRB-N); published Italian norms were used for correction of the raw scores (Amato et al. 2006). The BRB-N evaluates the following cognitive domains through different subtests (Table 2): verbal memory [through the 3 sub-scores of the Select Reminding Test: SRT- Long-Term Storage, cut-off = 23.3; Consistent Long-Term Retrieval, cut-off = 15.5; Delayed Recall, cut-off = 4.9]; visuo-spatial memory [Spatial Recall Test, cut-off = 12.7; Delayed Recall (SPART-D), cut-off = 3.6]; attention, processing speed and visual scanning [Symbol Digit Modalities Test, cut-off = 37.9]; processing speed, working-memory and sustained attention [3- and 2-s-intervals Paced Auditory Serial Addition Task, cut-off = 17.1 and 28.4, respectively]; semantic verbal fluency [Word List Generation; cut-off = 17.0].

Healthy controls did not undergo the neuropsychological assessment since they had no history of psychiatric or neurological illness.

Demographic and clinical features, including EDSS scores, of individuals affected by RRMS are reported in Tables 1 and 2.

**Table 1:** MS participants’ demographic and clinical data

Patient	Age (years)	Education (years)	Gender	Date of MS diagnosis	EDSS score	Drugs	Audiometry	Visual acuity and contrast sensitivity
#1	78	7	F	2009	6.5	Clonazepam, sativex, pregablin	n.a	Contrast sensitivity 80:20/125* 40: 20/50 1: 20/12.5
#2	41	18	F	1997	7	Baclofen	n.a	Contrast sensitivity 80:20/80* 40: 20/40* 1: 20/16
#3	43	13	F	1998	6	Vitamin C, paroxetine, nadroparin	n.a	Visual acuity: right eye = J3 nat left eye = J3 nat
#4	56	13	M	2003	6.5	Paroxetine, gabapentin, triatec, tavor	Normal	Contrast sensitivity 80:20/200* 40: 20/80* 1: 20/25*
#5	46	18	F	2002	6.5	Fampridine, setraline, oxybutynin	Normal	Contrast sensitivity 80: N.E 40: 20/125* 1: 20/25*
#6	46	8	F	2013	7	Simvastatin, glyatiramer, ketoprofene, clonazepam	Normal	Contrast sensitivity 80:20/80* 40: 20/32 1: 20/16
#7	41	10	F	2000	6	Paroxetine, baclofen, tizanidine, phingolimod, tolterodyne, nimesulid, psyllogel, glicerine	Normal	Contrast sensitivity 80:20/125* 40: 20/25 1: 20/20*
#8	45	16	M	1996	7	dexamethasone, Levothyroxine, calcium carbonate, cholecalciferol, zoledronate	Normal	Contrast sensitivity 80:20/80* 40: 20/32 1: 20/16
#9	34	10	F	2009	2.5	Natalizumab	n.a	n.a
#10	42	13	F	2002	1.5	No therapy	n.a	n.a
#11	54	8	F	2012	6.5	Dibase, melatonin	Normal	Contrast sensitivity 80:20/80* 40: 20/50* 1: 20/10
#12	49	12	F	2010	4	No therapy	n.a	n.a
#13	39	13	M	2015	4	No therapy	n.a	n.a
#14	65	18	M	2000	6.5	Levothyroxine, amlodipine, oxybutynin, delta-9-tetrahydrocannabinol and cannabidiol	n.a	Contrast sensitivity 80:20/200* 40: 20/125* 1: 20/25*
#15	52	8	F	1986	4.5	Theriflunomide	n.a	n.a
#16	43	13	F	2010	4	No therapy	n.a	n.a
#17	50	13	M	2012	6	Interferon beta-1a	n.a	n.a
#18	53	12	M	2003	6.5	ventolin, oxybutynin, phingolimod, omega-3	Mild bilateral hearing loss for acute frequencies	Contrast sensitivity 80:20/40 40: 20/125* 1: N.E
#19	41	18	M	1998	7	Targin, gabapentin, biotin, tysabri	n.a	Visual acuity: right eye = J12 csl left eye = J3 csl
#20	50	13	F	1986	6	Mesalazine, extavia (interferon beta-1b)	n.a	n.a
#21	63	18	F	1991	6.5	Vitamin D	n.a	n.a
#22	52	15	M	1998	8	Targin, gabapentin, fampyra, omeprazole, oxybutynin	n.a	n.a
#23	42	8	M	2010	1.5	Copolimer acetate	n.a	n.a
#24	54	18	M	2011	6	Fampridine, Ocrelizumab, baclofen, amlodipine	n.a	n.a
#25	49	8	F	2017	6	theriflunomide, baclofen, Gabapentin, lactulose, omeprazole	n.a	n.a

*M/F* male/female; *EDSS* expanded disability status scale (ranging from 0 to 10 in 0.5 unit-increments that represent higher levels of disability); *n.a.* not available

*Abnormal

**Table 2:** MS participants’ scores at Rao’s brief repeatable battery (BRB-N) subtests and at mini mental state examination (MMSE)

BRB-N subtests and MMSE											
Patient	Score	SRT-LTS	SRT-CLTR	SRT-D	SPART	SPART-D	SDMT	PASAT 3	PASAT 2	WLG	MMSE
#1	Raw	36	19	6	12	5	35	15	n.a	22	n.a
	Corrected	43.6	27.3	7.1	14	5.7	41.2	24.2*	n.a	19.9	n.a
#2	Raw	58	47	8	22	5	59	50	41	32	n.a
	Corrected	50.2	38.4	6.9	19.9	4.3	53.2	40.5	34.8	29.9	n.a
#3	Raw	63	51	12	14	8	57	34	26	26	29
	Corrected	62.2	50.1	12	13.8	7.9	56.4	33	25.3	23.9	27
#4	Raw	28	19	4	28	5	44	55	32	26	29
	Corrected	27.2	18.1	3.9*	27.8	4.9	43.4	54	31.3	28.1	26.8
#5	Raw	37	33	7	24	8	44	47	31	33	29
	Corrected	29.15	24.4	5.8	21.9	7.28	38.2	37.5	24.8	30.9	27
#6	Raw	56	42	8	19	4	42	55	21	26	30
	Corrected	62.2	48.8	8.9	20.6	4.6	46.5	60	25.9	28.1	26
#7	Raw	19	10	9	27	19	34	5	n.a	19	28
	Corrected	20.3*	12.15*	9.2	27.5	16.8	35.4*	7.4*	n.a	16.9*	27
#8	Raw	40	35	9	25	9	56	58	56	30	30
	Corrected	35	29.5	8.3	23.7	8.5	52.3	51.9	51.9	32.1	27
#9	Raw	57	35	6	21	6	52	51	38	29	n.a
	Corrected	60.4	38.7	6.5	21.9	6.3	54.4	55.1	40.7	26.8	n.a
#10	Raw	45	24	7	17	5	51	52	32	31	n.a
	Corrected	44.2	23.1	6.8	16.7	4.9	50.3	50.9	31.3	28.8	n.a
#11	Raw	38	34	5	19	7	40	39	35	25	30
	Corrected	44.2	40.8	5.9	20.6	7.6	44.5	46.5	39.9	22.9	25
#12	Raw	36	20	6	15	3	40	17	n.a	n.a	n.a
	Corrected	36.6	20.6	6.1	15.2	3.1*	40.4	17.7*	n.a	n.a	n.a
#13	Raw	23	7	3	14	5	37	29	n.a	16	n.a
	Corrected	22.2*	6.8*	2.9	13.6	4.6	36.5*	27.5*	n.a	18.1	n.a
#14	Raw	49	39	8	17	7	44	35	30	24	30
	Corrected	41.15	30.4	6.9	14.3	6.3	38.2	25.5*	23.8	26.1	27
#15	Raw	26	0	2	15	6	34	n.a	n.a	12	n.a
	Corrected	32.1	0*	2.9*	16.6	6.6	38.2	n.a	n.a	9.9*	n.a
#16	Raw	37	34	7	26	10	58	58	34	30	n.a
	Corrected	36.2	33.1	6.9	25.8	9.9	57.4	57	33.3	27.9	n.a
#17	Raw	9	9	6	16	5	18	n.a	n.a	10	n.a
	Corrected	8.2*	8.7*	5.8	15.8	4.9	17.4*	n.a	n.a	12*	n.a
#18	Raw	12	0	0	10	6	28	19	n.a	13	26
	Corrected	12.8*	0*	0*	10.2*	6.1	28.6*	20.02*	n.a	15.1*	27
#19	Raw	39	22	5	18	6	41	43	25	28	n.a
	Corrected	31.15	13.4*	3.9*	15.9	5.3	35.2*	33.5	18.8	30.1	n.a
#20	Raw	33	21	6	17	5	30	27	n.a	22	27
	Corrected	32.2	20.1	5.9	16.8	4.9	29.4*	25.9*	n.a	19.9	27
#21	Raw	31	19	9	14	6	48	16	n.a	23	30
	Corrected	23.15*	10.4*	7.9	11.9*	5.3	42.2	6.49*	n.a	20.9	26
#22	Raw	22	19	3	8	4	53	53	30	25	29
	Corrected	18.35*	14.99*	2.5*	7.04*	3.7	50.3	48.6	27.1	27.1	27
#23	Raw	31	28	8	12	4	32	45	28	12	n.a
	Corrected	37.2	34.8	8.9	13.6	4.6	36.5*	52.5	32.9	14.1*	n.a
#24	Raw	29	18	5	17	6	51	52	36	32	n.a
	Corrected	21.15*	9.4*	3.9*	14.9	5.3	45.2	42.5	29.8	34.1	n.a
#25	Raw	26	18	5	11	3	46	33	n.a	24	29
	Corrected	32.2	24.8	5.9	12.6*	3.6*	50.5	40.5	n.a	21.9	27
Counts (N)		25	25	25	25	25	25	23	15	24	13
Mean		40.95	31.44	7.08	18.61	6.36	46.82	46.93	31.44	26.18	26.67
Median		32.2	23.1	6.1	15.9	5.3	42.2	40.5	31.3	25	27
St. dev		11.22	9.87	1.88	4.67	2.73	6.45	8.57	8.22	4.56	0.62
Range		8.2–62.2	0–50.1	0–12	7.04–27.8	3.7–16.8	17.4–57.4	6.49–60	18.8–51.9	9.9–34.1	25–27

*SRT-LTS* select reminding test-long-term storage; *SRT-CLTR* select reminding test-consistent long-term retrieval; *SRT-D* select reminding test-delayed recall; *SPART* spatial recall test; *SPART-D* spatial recall test-delayed recall; *SDMT* symbol digit modalities test; *PASAT 3/2* 3/2 s-intervals paced auditory serial addition task; *WLG* word list generation; *MMSE* mini mental state examination; *St. dev.* standard deviation; *n.a.* not available

*Pathological score (performance below the cut-off)

### Simultaneity judgment task (SJ2)

Participants underwent 3 versions of the SJ2 task: bimodal audio-visual, unimodal auditory and unimodal visual SJ2; the unimodal versions of the task represented our control tasks. The three SJ2 tasks were given in a counterbalanced order (i.e., ABC-BCA-CBA) between subjects and in different sessions separated by at least 4 h (maximum interval = 24 h).

In the SJ2 task, visual and auditory stimuli were presented, respectively, on an LCD PC screen and by two loudspeakers placed on each side of the PC screen. The visual stimulus was a white ring (diameter = 9.4 cm; duration = 30 ms) presented at the center of the screen on a uniform black background, which was aligned with the midsagittal plane of the participant’s trunk; the auditory stimulus was a pure tone of 3500 Hz (duration = 30 ms). The presentation of both stimuli was followed by the appearance of a white fixation cross at the center of the black screen for 2000–3000 ms (randomized inter-trial interval).

**Fig. 1:**
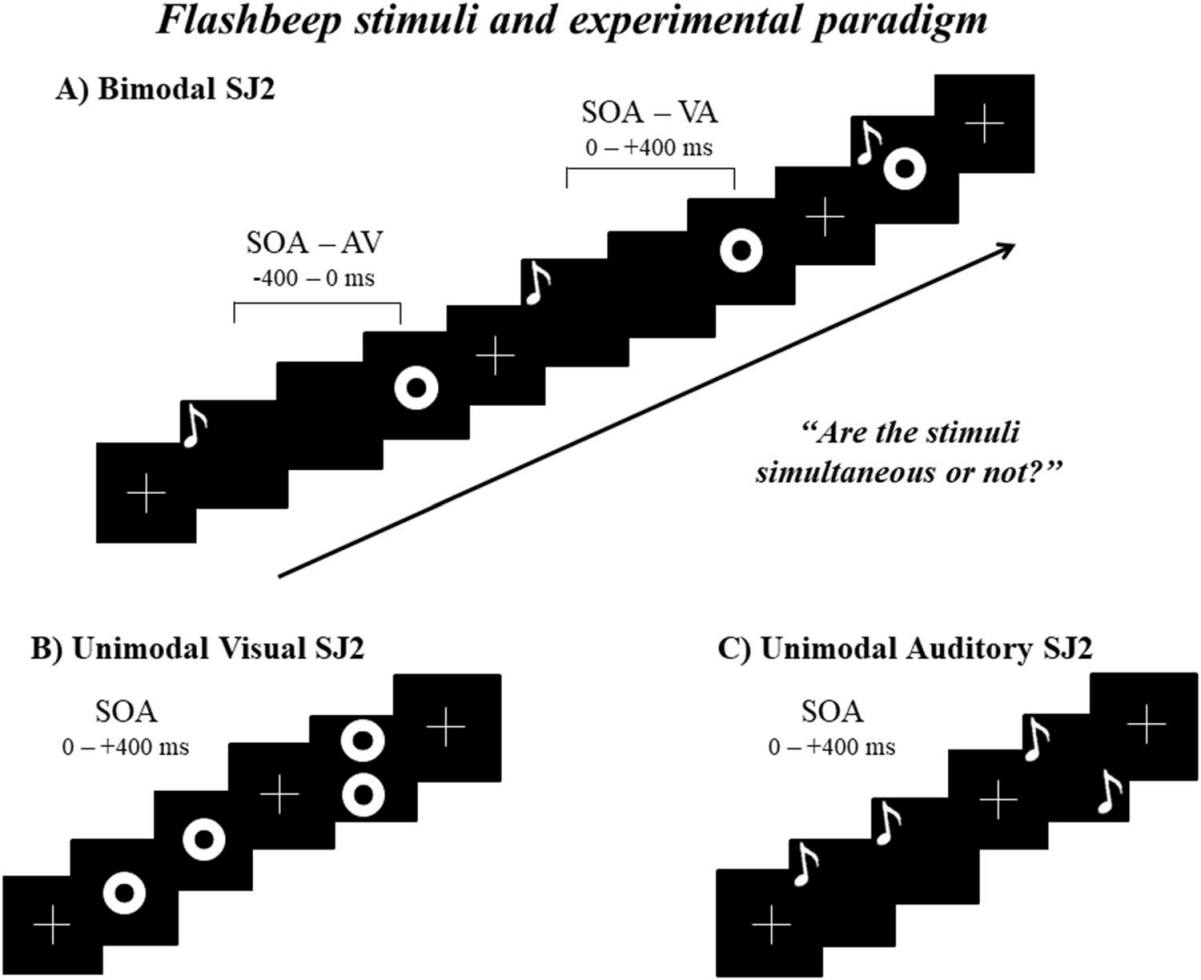
Experimental trials. Examples of experimental trials in the SJ2 task, during which pairs of stimuli with various stimulus onset asynchrony (SOA; 17 SOAs = 0, ± 50, ± 100, ± 150, ± 200, ± 250, ± 300, ± 350, ± 400 ms;—means auditory first, + visual first) were presented. (**A**) In the bimodal SJ2 task, an Auditory stimulus (**A**) was presented with a Visual stimulus (V); AV = auditory first (SOAs with negative values), VA = visual first (SOAs with positive values). (**B**, **C**) In the unimodal SJ2 task, 2 visual or 2 auditory stimuli were presented with 9 SOAs (from 0 to 400 ms)

During the task, pairs of unimodal (visual or auditory) or bimodal (audio-visual) stimuli were presented simultaneously or separated by different SOAs. In the audio-visual bimodal SJ2 (see Fig. 1), the following 17 SOAs were used: 0, ± 50, ± 100, ± 150, ± 200, ± 250, ± 300, ± 350, ± 400 ms (- means auditory first, + visual first). For each SOA, 20 trials were given (total trials = 340; task duration ~ 30 min) in randomized order. In both unimodal SJ2 tasks (visual and auditory), 9 SOAs were used: 0, 50, 100, 150, 200, 250, 300, 350, 400 ms. For each SOA, 20 trials were given (total = 180; task duration =  ~ 15 min) in randomized order. Each experimental block was divided into 2 sub-blocks to allow a possible break according to the participant’s fatigue level. The whole experiment lasted approximately 1 h. Stimulus presentation and response recording were under computer control (E-Prime Software, Psychology Software Tools Inc., Pittsburgh, PA).

During the task, participants sat comfortably in an armchair in front of the PC screen (distance = 60 cm), in a dimly illuminated room. In each trial, participants were required to report, as accurately and fast as possible, whether the 2 stimuli presented were simultaneous (**“**YES**”** response) or not (**“**NO**”** response) (i.e., 2-forced-choice task). Healthy participants responded with their dominant hand by pressing the corresponding button of the PC keyboard; response buttons were counterbalanced between participants so that for half of them the up-arrow corresponded to the **“**YES**”** response, while the down-arrow to the **“**NO**”** response, and for the other half the opposite. In light of possible motor difficulties and slowness due to subclinical dexterity impairment, individuals with RRMS were required to give a verbal response in each trial, and the corresponding response button was pressed by the experimenter. There was no time limit for responding regardless of the response modality; only after the answer had been given, the new trial started.

Before starting the experimental session, participants underwent a practice session to familiarize with the task and to verify its comprehension. During the practice session, 2 trials were given for each of following SOAs: 0, ± 100, ± 200, ± 300, ± 400 ms; at the end of each trial, subjects received feedback about their response accuracy. Instead, no feedback was given during the experimental session.

### Statistical analysis

#### Sample size estimation

The sample size was computed by means of a power analysis (Faul et al. 2009), which showed that a sample size of 16 participants for each group was proper for detecting the effects of interest considering an effect size of 0.80 (Alpha Error Level: *p* = 0.05; Statistical Power, i.e., 1-Beta = 0.95). Choosing a more conservative 0.01 criterion of statistical significance (considering possible corrections for multiple comparisons), a sample size of 22 participants was estimated. Opting for this safest choice, and also considering possible drop-outs, 25 participants with RRMS were enrolled in the present study. This RRMS sample size is broadly in line with that of previous studies in the field of experimental neuropsychology (*N* =  ~ 15/ ~ 25; e.g., Bolognini et al. 2012, 2016; Ronchi et al. 2009).

### Bimodal SJ2 task

**Fig. 2:**
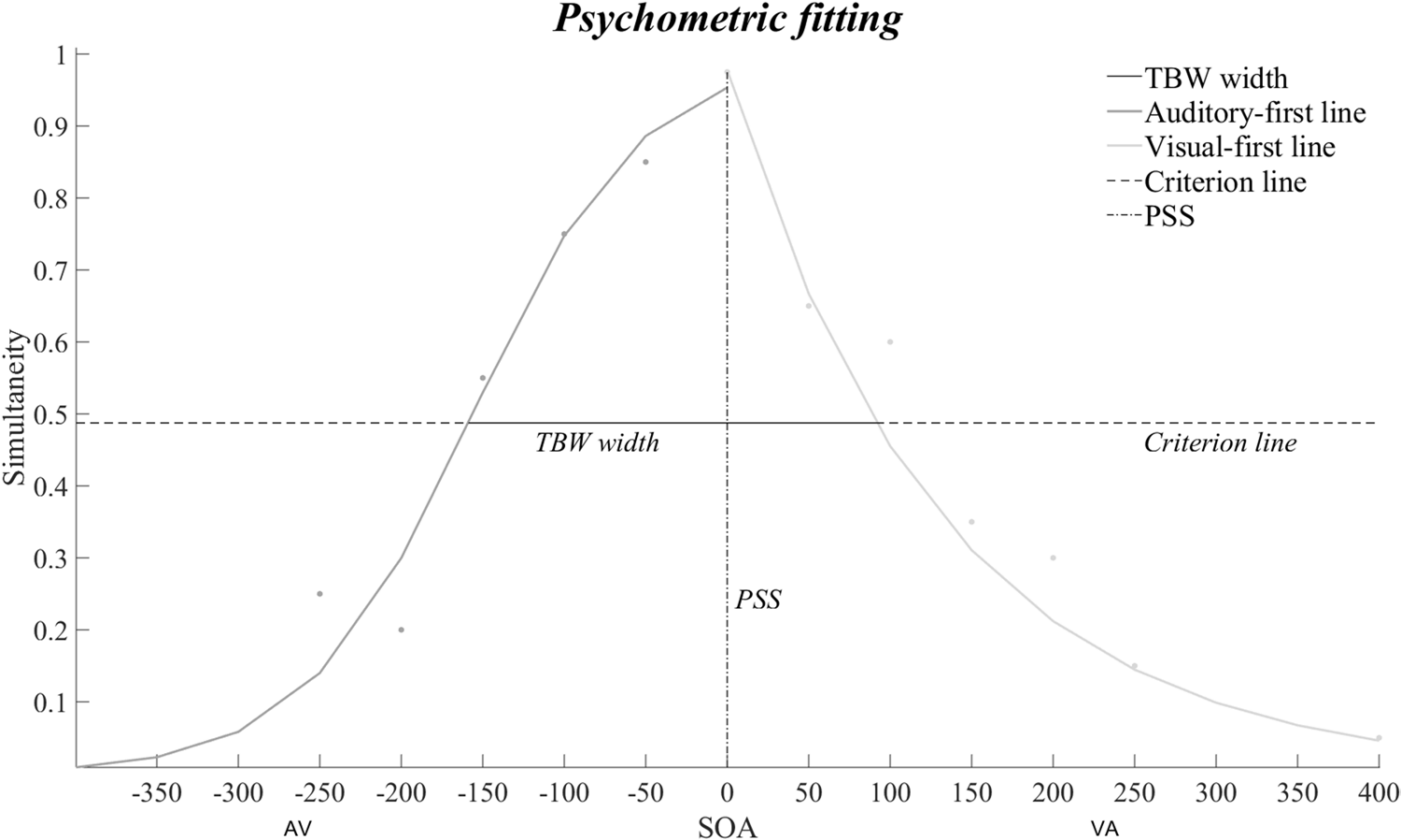
Bimodal SJ2 task. Psychometric fitting of a representative subject

The multisensory TBW was measured separately for each group as following. First, to calculate the individual TBW, the proportion of simultaneity responses at every SOAs was computed (i.e., participants’ reports of perceived audio-visual synchrony). For each participant, the distribution of such responses across SOAs was then split into the left and right sides of the window, and separately fitted to a psychometric sigmoid and logarithmic function, referred to the left and right sides of the windows, which correspond to auditory-first presentation and visual-first presentation, respectively. These best-fit functions were computed by using the *glmfit* function implemented in MATLAB (Stevenson et al. 2012). These data were used to create two best-fit curves, both including the temporal simultaneity (0 ms SOA) condition. A criterion according to which measuring the width of the window was established (i.e., *criterion line*), which was set at half the distance between the mean individuals’ lowest and highest simultaneity perception (about 50% perception of simultaneity; Powers et al. 2009). A parameter derived from this curve is its width, that reflects the TBW extension and corresponds to the SOAs range at which the two sensory stimuli, auditory and visual, are perceived as simultaneous (Stevenson et al. 2012; Vroomen and Keetels 2010). Participants’ auditory-first and visual-first TBWs were estimated as the SOA ranges at which the best-fit curve’s *y* value intercepted the criterion line. The peak of this curve corresponds to the PSS, which denotes the SOA along the x-axis where the two different sensory stimuli are perceived as being maximally simultaneous. Therefore, in the SJ2 task, the PSS represents the interval at which the participants perceive the incoming inputs as simultaneous, while the TBW represents the range of tolerance within which participants perceive synchrony (Vatakis et al. 2018). The distribution width was computed for both the left and the right side of the window (i.e., from the left-most point at which the curve intersected the criterion line to zero, and from zero to the right-most point at which the curve crossed the criterion line), and then combined to get an estimation of the total distribution width (see Fig. 2 for an example of psychometric fitting of the data of a representative participant).

Normality distribution of data was assessed by means of Shapiro–Wilk test. When normally distributed, group differences in TBW width were analyzed using independent sample *t* tests. When TBW values violated normality (Shapiro–Wilk test, *p* < 0.05), TBW width were log-transformed to obtain a normal distribution and, accordingly, parametric statistics were run. When TBW data distribution violated normality even after log-transformation, the non-parametric Mann–Whitney *U* test was adopted. The same statistical approach used to analyze TBW values was adopted to examine group differences (i.e., RRMS participants vs. healthy controls) in PSS for the bimodal SJ2 task.

Pairwise effect sizes were expressed by means of Cohen’s *d* (as regard the parametric pairwise comparisons) and by rank biserial correlation (as regard the non-parametric pairwise comparisons).

Perception of audio-visual simultaneity across SOAs (i.e., bimodal SJ2) was further analyzed by a repeated-measures ANOVA on the mean reported perception of simultaneity with Group (individuals with RRMS, healthy controls) as between-subject factor and SOA (from − 400 to + 400 ms) as within-subject factor.

The magnitude of ANOVA effect size was expressed by *η*_*p*_^2^. Post hoc comparisons were performed by means of Bonferroni test.

### Unimodal control SJ2 tasks

The control unimodal (visual and auditory) SJ2 tasks were analyzed by using the same ANOVA model adopted for the bimodal SJ2 task, with the additional factor Modality (visual and auditory). Thus, simultaneity perception in unimodal SJ2 tasks was analyzed by a repeated-measures ANOVA on the mean simultaneity perception responses with Group (individuals with RRMS, healthy controls) as between-subject factor and Modality (visual or auditory) and SOA (from − 400 to + 400 ms) as within-subject factors.

As for the ANOVA computed for the bimodal SJ2 task, the magnitude of effect size was expressed by ηp^2^. Post hoc comparisons were performed by means of Bonferroni test.

### Correlational analyses

Finally, RRMS participants’ scores at the clinical tests were computed by means of Pearson’s correlation coefficient in order to explore possible associations between multisensory TBW width and neuropsychological scores.

For all statistical analysis (i.e., TBW, ANOVAs on bimodal and unimodal SJ2 tasks, correlational analyses), statistical significance was set at *p* value < 0.05.

## Results

First, the two experimental groups (participants with RRMS vs. healthy controls) were compared by means of independent *t* tests to detect possible differences with respect to age and level of education; results showed that the two groups were comparable for both variables: age, *t*_53_ = 0.007, *p* = 0.99; level of education, *t*_53_ = 0.34, *p* = 0.73.

### Bimodal SJ2 task

All participants (i.e., 25 individuals affected by RRMS and 30 healthy controls) completed the task as described. For participants affected by RRMS, TBW values were normally distributed (Shapiro–Wilk test, *p* = 0.28), while TBW values violated normality in the control group (Shapiro–Wilk test, *p* = 0.002); hence these data were log-transformed to obtain a normal distribution (Shapiro–Wilk test, *p* = 0.55). Accordingly, parametric statistics were adopted.

**Fig. 3:**
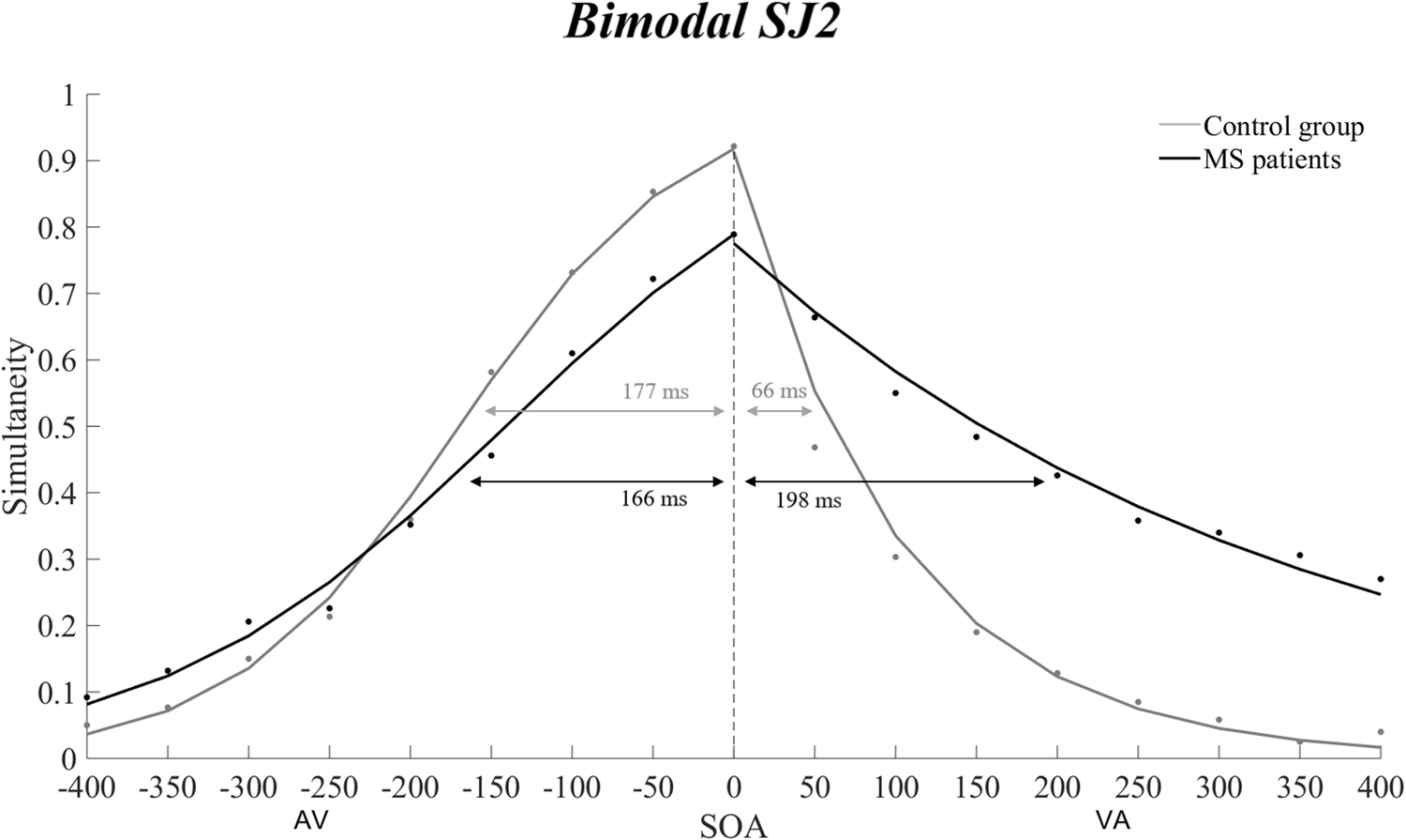
Results from the bimodal SJ2 task. Participants affected by RRMS (black line and arrow) show an enlarged audio-visual TBW (from − 166 to + 198 ms; − means auditory stimulus first, + visual first), as compared to healthy controls (grey line and arrow; from − 177 to + 66 ms). Higher proportion of simultaneity perceptions were detected when the visual stimulus preceded the auditory stimulus by + 100 ms to + 350 ms, as compared to healthy controls (all *ps* < 0.03)

Results showed an enlarged total multisensory TBW in participants with RRMS (364.22 ms ± 136.18), as compared to healthy controls (242.78 ms ± 103.11; *t*_53_ = 3.08; *p* = 0.003; Cohen’s *d* = 0.84; see Fig. 3). With respect to the left side of TBW, data of both groups were normally distributed (all *p*s > 0.09), and participants with RRMS (− 165.64 ms ± 88.91) did not differ from healthy controls (− 177.22 ms ± 59.20; *t*_53_ = 0.70; *p* = 0.48; Cohen’s *d* = 0.19). The right-sided TBW values violated normality even after log-transformation (*p* = 0.006). The non-parametric Mann–Whitney *U* test showed an enlarged right TBW in participants with RRMS (+ 197.57 ms ± 206.63), as compared to healthy controls (+ 65.56 ms ± 87.88; *U* = 204, *z* = 2.88, *p* = 0.003; rank biserial correlation = 0.46; see Fig. 3). Overall, fitting results documented an asymmetrically enlargement of the multisensory TBW in participants with RRMS, as compared to healthy controls.

**Fig. 4:**
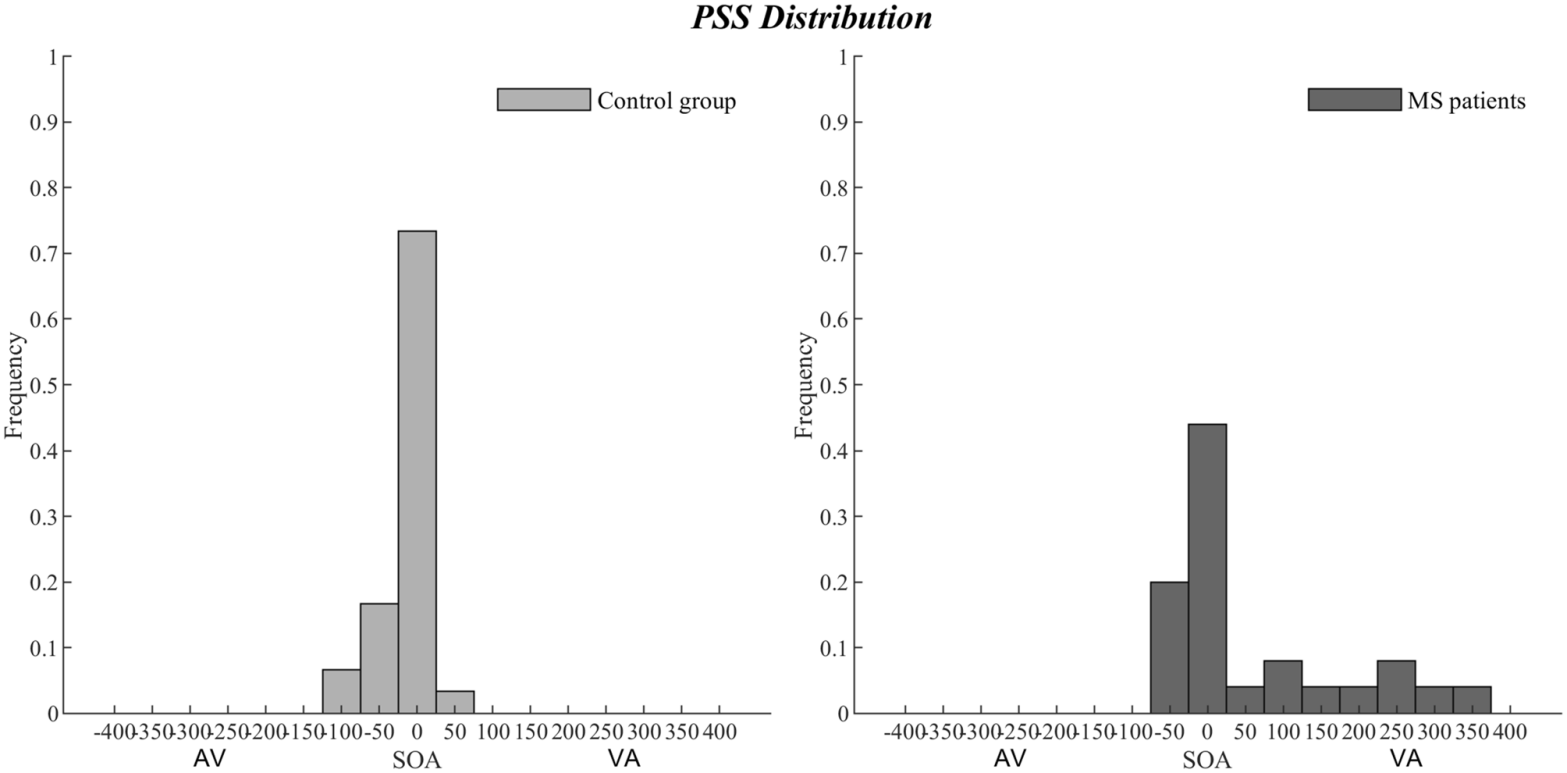
Point of subjective simultaneity (PSS) distribution in the bimodal SJ2 task. As compared to healthy controls (left panel), participants affected by RRMS (right panel) show a greater variability with respect to the number of SOAs where the visual and auditory stimuli were perceived as simultaneous

Since values violated normality (Shapiro–Wilk test, *p* < 0.001 for both groups), PSS differences between RRMS and healthy participants at the bimodal SJ2 task were computed by means of Mann–Whitney *U* test, which showed a different variability concerning the SOAs within which audio-visual stimuli were perceived simultaneous in healthy controls (0 ms ± 31.98; SOA range: from − 100 to 50 ms) and in individuals affected by RRMS (0 ms ± 119.89; SOA range: from − 50 to + 350 ms; *U* = 260, *z* = 2.19, *p* = 0.02; rank biserial correlation = 0.31; see Fig. 4).

The ANOVA showed significant main effects of Group [*F*_1,53_ = 9.5027, *p* = 0.003, *η*_*p*_^2^ = 0.15] and of SOA [*F*_16,848_ = 85.288, *p* < 0.001, *η*_*p*_^2^ = 0.62], and a significant Group X SOA interaction [*F*_16,848_ = 10.400, *p* < 0.001, *η*_*p*_^2^ = 0.16]. The significant main effect of the factor Group was qualified by higher perception of simultaneity in participants with RRMS as compared to healthy controls (41% ± 0.025 vs. 31% ± 0.022). The significant main effect of the factor SOA was mainly qualified by higher proportion of simultaneity perceptions at SOAs of 0 (86% ± 0.019) and − 50 (79% ± 0.026) ms, as compared to the other SOAs conditions (all ps < 0.001). The significant Group X SOA interaction was explained by the enlarged right side of the multisensory TBW in participants with RRMS, as compared to healthy controls; in particular, individuals with RRMS presented higher levels of simultaneity perception as compared to controls at all SOAs from + 100 to + 350 ms (all *ps* < 0.03).

No significant correlations emerged between the multisensory TBW width and scores at each BRB-N test of RRMS participants (all ps > 0.98).

The absence of differences between the two response modalities (verbal responses required to participants with RRSM vs. manual responses for the healthy controls) was then checked by administering the bimodal SJ2 task to 12 new healthy participants, with a mean age and a level of education similar to that of the participants affected by SM (mean = 43.92 ± 12.89; years of education = 15.5 ± 3.63). Half of these healthy participants (*N* = 6) performed the bimodal SJ2 task giving verbal responses, while the other half gave manual responses. The two groups of participants did not differ with respect to age and level of education (all *ps* > 0.38). Results from the repeated ANOVA, with Response modality (verbal vs. manual response) as between-subject factor and SOA as within-subject factor, on the mean rate of simultaneity perception did not show significant effects of the Response modality (*p* = 0.9) and its interaction with SOA (*p* = 0.9). As regard the psychometric fitting, results showed no difference in the total TBW between the verbal response group (237.4 ms ± 138.5) and the manual response group (236.99 ms ± 110.8, *p* = 0.98). Also, the PSS analysis did not show variability differences concerning SOAs of simultaneity perception between the two groups (*p* = 0.5).

### Unimodal control SJ2 tasks

**Fig. 5:**
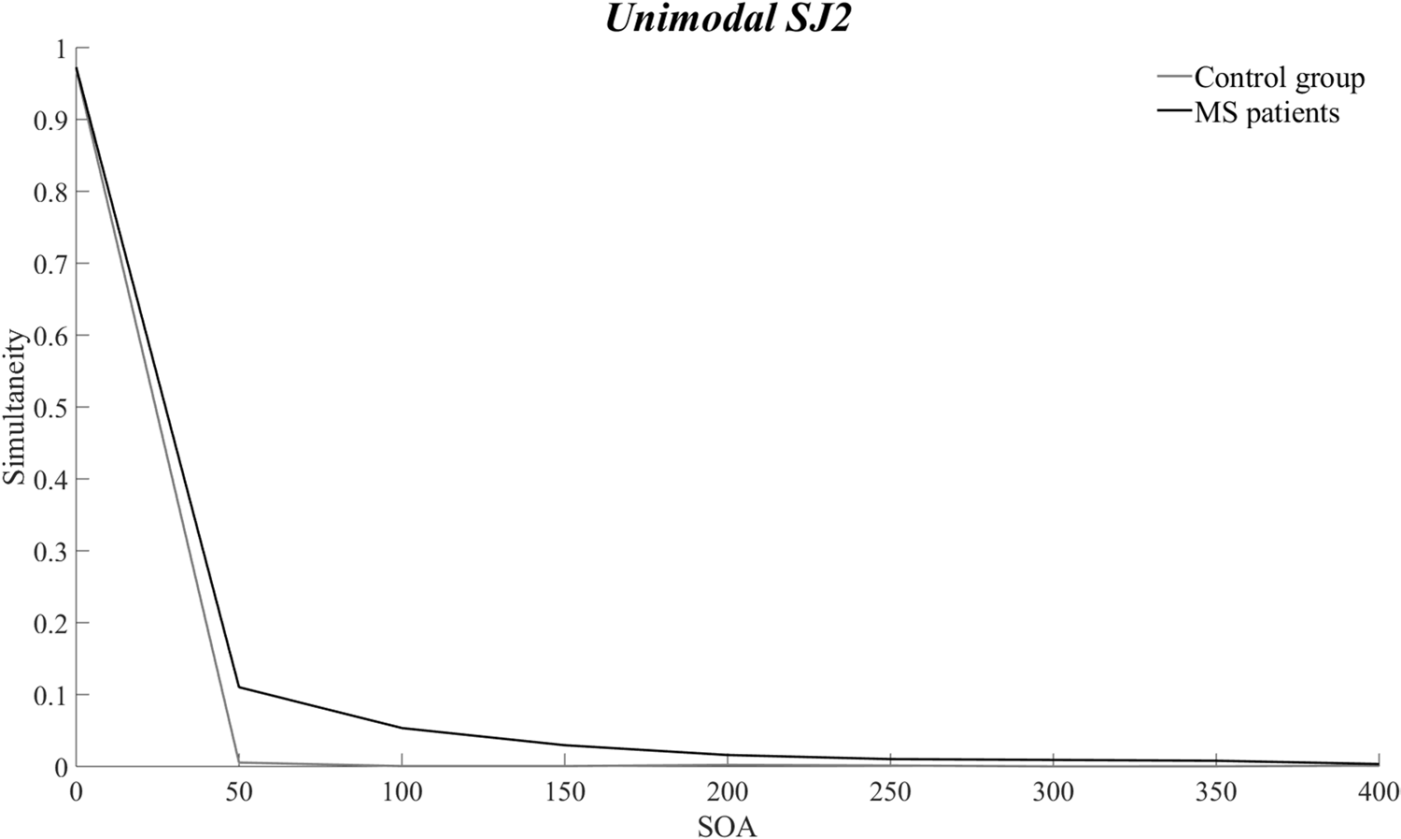
Results from the unimodal SJ2 tasks. Unimodal (mean responses at the visual and auditory tasks) simultaneity perception differs between RRMS (black line) and healthy participants (grey line) only when stimuli are presented at the very short SOA of 50 ms (*p* < 0.001), with participants affected by RRMS being more prone to report simultaneity with such short delay

Three out of 25 participants affected by RRMS were excluded from statistical analyses since they could not complete the unimodal tasks, due to their discharge from the clinic. Hence, 22 individuals with RRMS and 30 healthy controls were considered in the statistical analyses of the unimodal tasks. Results of the ANOVA showed significant main effects of the Group [*F*_1,50_ = 6.1616, *p* = 0.02, *η*_*p*_^2^ = 0.11], with higher proportion of simultaneity perception responses in participants with RRMS as compared to controls (13% ± 0.008 vs. 11% ± 0.007), as well of the SOA [F_8,400_ = 1691.6, *p* < 0.001, *η*_*p*_^2^ = 0.97], with higher levels of simultaneity perception with 0 and 50 ms of SOA (respectively: 97% ± 0.006; 5.7% ± 0.02) as compared to almost all the other SOAs (all *ps* < 0.02). Simultaneity perception between visual and auditory modality did not differ (main effect of Modality: *p* = 0.26). The significant Group X SOA interaction [*F*_8,400_ = 4.7311, *p* < 0.001, *η*_*p*_^2^ = 0.08] showed higher levels of simultaneity perception in RRMS as compared to healthy controls at 50 ms of SOA (11% ± 0.03 vs. 0.5% ± 0.02, respectively; *p* < 0.001; see Fig. 5). The interactions Group X Modality (*p* = 0.23), Modality X SOAs (*p* = 0.41), Group X Modality X SOAs (*p* = 0.45) did not reach significance.

### Correlational analyses

Correlational analyses were computed considering all the 25 participants with RRMS. A moderate negative correlation between RRMS participants’ score at SPART test and TBW width was found (Pearson’s *r* = − 0.47, *p* = 0.02); no significant correlation was found between multisensory TBW width and scores at all the other neuropsychological tests in participants affected by RRMS (all *ps* > 0.08); moreover, a weak correlation was found between TBW width and EDSS scores (Pearson’s r = − 0.39, *p* = 0.05; see Table 3).

**Table 3:** Correlational analyses between MS participants’ score at Rao’s brief repeatable battery (BRB-N) subtests, mini mental state examination (MMSE), expanded disability status scale (EDSS) and the width of the multisensory temporal binding window (TBW)

		Entire TBW	Right side TBW	Left side TBW
SRT-LTS	Pearson’s *r* *p* value	0.108 0.607	0.044 0.833	-0.090 0.670
SRT-CLTR	Pearson’s *r* *p* value	− 0.219 0.293	− 0.213 0.307	− 0.029 0.892
SRT-D	Pearson’s *r* *p* value	− 0.194 0.353	− 0.265 0.201	− 0.155 0.459
SPART	Pearson’s *r* *p* value	− 0.468* 0.018	− 0.246 0.236	0.296 0.151
SPART-D	Pearson’s *r* *p* value	− 0.354 0.082	− 0.369 0.070	− 0.087 0.678
SDMT	Pearson’s *r* *p* value	− 0.123 0.559	− 0.068 0.748	0.072 0.732
PASAT 3	Pearson’s *r* *p* value	− 0.163 0.457	0.021 0.922	0.265 0.222
PASAT 2	Pearson’s *r* *p* value	− 0.260 0.348	− 0.306 0.268	− 0.144 0.609
WLG	Pearson’s *r* *p* value	− 0.219 0.303	− 0.063 0.769	0.251 0.237
MMSE	Pearson’s *r* *p* value	0.456 0.118	0.221 0.469	− 0.336 0.262
EDSS	Pearson’s *r* *p* value	− 0.399* 0.048	− 0.513* 0.009	− 0.265 0.200

*SRT-LTS* select reminding test-long-term storage; *SRT-CLTR*  select reminding test-consistent long-term retrieval; *SRT-D* select reminding test-delayed recall; *SPART* spatial recall test; *SPART-D* spatial recall test-delayed recall; *SDMT* symbol digit modalities test; *PASAT 3/2* 3/2 s-intervals paced auditory serial addition task; *WLG* word list generation; *MMSE* mini mental state examination; *EDSS* expanded disability status Scale

*Significant correlation

## Discussion

In this study, we aimed at exploring the presence of possible alterations in multisensory integration in individuals affected by RRMS to verify whether this disease may compromise the ability to bind incoming auditory and visual stimuli depending on their temporal relationship. With this intention, we measured the width of the multisensory TBW in RRMS (comparing it to that of neurological healthy controls) as it represents a reliable marker of the temporal range at which the brain tolerates asynchronies in incoming stimuli from different sensory modalities (here, visual and auditory).

The present findings demonstrate an abnormal multisensory TBW in RRMS, in face of a normal temporal processing of unisensory visual and auditory information. Specifically, healthy participants reported to perceive that the auditory and visual stimuli were simultaneous over a larger inter-stimulus interval of approximately 243 ms. In particular, audio-visual simultaneity was perceived by healthy participants with SOAs ranging from 0 to − 177 ms when the auditory stimulus precedes the visual one, and with SOAs ranging from 0 to + 66 ms when the visual stimulus comes first. In line with previous evidence in humans **(**Conrey and Pisoni 2006; Stevenson et al. 2012; Wallace and Stevenson 2014), multisensory stimuli with a hundred (or more) milliseconds of delay are typically perceived as temporally coincident, hence showing a degree of tolerance for temporal asynchrony for audio-visual interactions (Conrey and Pisoni 2006; Wallace and Stevenson 2014). Instead, RRMS participants’ multisensory interactions take place over a broader TBW, in particular when the visual stimulus precedes the auditory ones. Indeed, individuals affected by RRMS report perceiving audio-visual simultaneity over a large asymmetrical interval of ~ 364 ms (i.e., temporal range from − 166 to 0 ms when the sound comes first, and from 0 to + 198 ms with the visual stimulus as first). The enlarged TBW in RRMS is specific for multisensory events, since it is within a normal range in the case of pairings of unimodal (visual or auditory) stimuli. This dissociation suggests a selective multisensory deficit of temporal acuity in RRMS. Therefore, there is an abnormal, enlarged, TBW in RRMS, in face of an overall normal ability to detect synchrony between events of the same sensory modality. This result indicates that in RRMS there is aberrant binding of multisensory information in the temporal domains.

Of interest is also the different variability of the PSS in healthy and RRMS participants. The PPS represents the temporal delay at which participants are more likely to perceive visual and auditory stimuli as synchronous during the bimodal SJ2 task. The PSS was constantly around 0 ms of SOA in healthy participants (range = − 100 to  + 50 ms), but highly variable in participants with RRMS, ranging between − 50 and + 350 ms (Fig. 4).

By itself, a general slowness of sensory processing featuring MS (Karussis 2014) could account for the observed multisensory deficit. However, we did not find such an impaired simultaneity perception for unimodal stimuli: despite an enlarged multisensory TBW, unimodal visual and auditory perception was largely unaffected in MS. Indeed, unimodal simultaneity perception differs between MS and healthy participants only when stimuli are presented at the very short SOA of 50 ms, with participants affected by RRMS being more prone to report simultaneity with such short delay. This finding seems to suggest a mild difficulty in segregating unimodal stimuli presented in fast sequence, but it cannot account for the multisensory deficit, given the much larger number of affected SOAs (from + 100 and + 350 ms) in the bimodal SJ2 task, in particular for the visual-first/auditory-second stimulus sequence (i.e., the right side of the TBW).

The dissociation between multisensory and unisensory simultaneity perception suggests that the enlarged multisensory TBW in participants affected by RRMS could result from a specific impairment of multisensory interactions at the neural level: the degenerative white matter lesions of MS may interrupt the transmission and interaction of information across the primary sensory cortices, or within higher order association brain areas (Compston and Coles 2008; Lubetzki and Stankoff 2014), in turn preventing the efficient integration of multisensory information within an optimal time window, while leaving unaltered the local (i.e., within the primary sensory cortex) temporal matching of modality-specific information.

In particular, the demyelination processes and axonal damage featuring the MS (Benedict et al. 2020) slow and even interrupt input transmission between brain areas (Denney et al. 2011; Litvan et al. 1988; Lubetzki and Stankoff 2014). To preserve optimal multisensory integration mechanisms, it is essential that information from different sensory modalities is efficiently and quickly transmitted across modality-specific sensory areas, as well as from unisensory to multisensory areas (Bolognini et al. 2013). Disturbances in communication between sensory brain areas may be responsible of the enlargement of the TBW width observed in our study.

From the perspective of a network approach, recently applied to the study of MS, multisensory deficit in the temporal domain could be also related to changes in modular organization in MS. A breakdown of functional modules in turn may lead to formation of larger modules, which is often observed in MS patients (Tahedl et al. 2018). Moreover, in MS, the occurrence of lesions in any part of the central nervous system likely causes local breakdowns affecting the binding of sensory information at different level of the sensory processing (Lubetzki and Stankoff 2014). Therefore, structural and/or functional connectivity damages, by disrupting the communication between sensory systems, represent a likely neurophysiological basis of the enlargement of the multisensory TBW in MS.

Abnormal multisensory integration processes in the temporal domain may have a cascade of consequences. The inability to distinguish which events in the environment are related or not based on the temporal structure of a multisensory stimulus pair reduces the availability of probabilistic information as to the sources of sensory information, in turn impacting on the ability to perceive the world in an accurate and meaningful way. Indeed, only within an optimal TBW the combination of information from different modalities can promote significant neural, behavioral and perceptual gains (Stein et al. 1993). For instance, as shown in some neuropsychiatric diseases such as dyslexia (Wallace and Stevenson 2014), autism spectrum disorders (Bebko et al. 2006; de Boer-Schellekens et al. 2013; Foss-Feig et al. 2010; Kwakye et al. 2011; Stevenson et al. 2014; Wallace and Stevenson 2014) and schizophrenia (Hahn et al. 2014; Stekelenburg et al. 2013; Wallace and Stevenson 2014), deficits of multisensory integration, by affecting perception and recognition of complex stimuli due to increased ambiguity about stimulus identity, negatively impact on high-level cognitive functions, among which language (Bebko et al. 2006; de Boer-Schellekens et al. 2013; Foss-Feig et al. 2010; Kwakye et al. 2011; Stevenson et al. 2014), frontal-executive functioning and visuo-spatial processing (Karussis 2014), the last shown to be impaired in MS too (Karussis 2014). Moreover, the ‘perceptual incoherence’ resulting from defective multisensory integration may give rise to incoherent self-experiences including depersonalization, ambivalence, diminished sense of agency and ‘loosening of associations’ between thoughts (Postmes et al. 2014). It is, therefore, plausible to hypothesize that multisensory integration deficits may worsen the cognitive functioning of RRMS patients, influencing even the psychological well-being and functional disability in this disease.

There are some limitations of the present study that should be considered. First, future studies should further explore the link between the TBW width and MS clinical symptoms. In the present study the small sample size and the absence of an in-depth clinical and neuropsychological examination of the participants with RRMS do not allow definitive conclusions to be drawn. For instance, we did not find any associations between the width of the multisensory TBW and cognitive functioning in RRMS, at least when assessed with the BRB-N. However, the BRB-N is a rapid screening battery for working-memory, some components of attention, information processing speed, and verbal memory, but it is insensitive to impairments in other cognitive domains. In this regard, it is also noteworthy that the onset of MS in our sample was heterogenous, ranging from 2 to 32 years, hence implying differences in the cognitive and neurological profiles. Moreover, the absence of an electrophysiological clinical evaluation of unimodal sensory processing (such as visual, short-latency somatosensory and short-latency brainstem auditory evoked potentials) may possibly have led to an underestimation of subclinical deficits responsible of subtle visual and auditory impairments unnoticed in our participants with RRMS. Although such hypothesis seems improbable since our findings showed no modality-specific (unimodal, visual and auditory) simultaneity perception deficits in RRMS participants, the availability of electrophysiological data could provide some further insight on the neurophysiological substrate about multisensory processing in MS.

In conclusion, the present study provides first evidence of impaired multisensory integration in RRMS. Future research should investigate whether such impaired multisensory integration also affects other phenotypes of MS, such as primary- and secondary-progressive MS. The investigation of multisensory integration in this disease is of relevance not only as model for studying the role of effective connectivity in multisensory perception, but also for clarifying the clinical role of multisensory abilities in MS, which could also encourage therapeutic interventions targeting multisensory disfunctions (Bolognini et al. 2013, 2015). It has been shown that multisensory impairments can be treated with behavioral and neurostimulation interventions (Feldman et al. 2020; Hamilton et al. 2013). Thus, a challenge could be to explore the chance of developing novel therapies for MS, targeting impaired multisensory processing, also assessing their effect on clinical (sensorimotor and cognitive) symptoms of MS. For instance, a growing body of evidence support the use of non-invasive brain stimulation to improve sensorimotor and cognitive functions in different neurological conditions through the improvement of network efficiency (Lefaucheur et al. 2020). On the other hand, there is some evidence supporting the importance of restoring multisensory integration to ameliorate cognitive and sensory disorders in neurological patients (Bolognini et al. 2013; Theves et al. 2020). With respect to the multisensory TBW, it has been shown that multisensory perceptual trainings can result in substantial alterations in the circuits underlying the perception of audio-visual simultaneity, in turn narrowing the multisensory TBW (Powers et al. 2009). This kind of evidence suggests a high degree of flexibility in multisensory temporal processing with implications for interventional strategies that may be used to ameliorate clinical conditions, such as MS, in which multisensory temporal function is impaired. Further investigations will be of relevance for clarifying the role of multisensory integration in MS pathophysiology and its impact on clinical symptoms and functional disability.

## Data Availability

Data and materials are available from the corresponding authors upon reasonable request.
